# Impact of bisoprolol transdermal patch on early recurrence during the blanking period after atrial fibrillation ablation

**DOI:** 10.1002/joa3.12538

**Published:** 2021-05-04

**Authors:** Yuya Suzuki, Masaru Kuroda, Tomoo Fujioka, Masayuki Kintsu, Tsubasa Noda, Akinori Matsumoto, Masahito Kawata

**Affiliations:** ^1^ Department of Cardiovascular Medicine Akashi Medical Center Akashi Hyogo Japan

**Keywords:** ablation, atrial fibrillation, bisoprolol transdermal patch, early recurrences, β‐blocker

## Abstract

**Background:**

Early recurrences of atrial arrhythmias (ERAAs) after ablation may require therapeutic intervention. The optimal medical therapy that prevents ERAAs requires clarification. This study aimed to compare the incidence of ERAAs between patients who received or did not receive bisoprolol transdermal patches (BTPs) at 3 months postablation.

**Methods:**

This single‐center retrospective study enrolled 203 consecutive patients with paroxysmal atrial fibrillation (AF) who had undergone their first ablation, comprising 59 in the BTP group and 144 in the non‐BTP group. Follow‐up assessments were conducted monthly for 3 months. We evaluated the incidence of ERAAs.

**Results:**

During the initial 1‐week observational period, the rate of ERAAs was lower in the BTP group (5.0%) than that in the non‐BTP group (18.8%) (*P* = .013). At 3 months postablation, the rate of ERAAs was lower in the BTP group (6.8%) than that in the non‐BTP group (25.7%) (*P* = .002). The cumulative freedom from ERAAs was significantly lower in the BTP group than in the non‐BTP group (log‐rank: *P* = .003). Administering BTPs was an independent factor that protected against ERAAs (odds ratio 0.181, [95% confidence interval 0.059‐0.559], *P* = .003).

**Conclusion:**

BTPs may prevent ERAAs after ablation.

## Graphical abstract

The patients who were administered bisoprolol transdermal patches (BTPs) immediately after atrial fibrillation (AF) ablation showed significant reductions in the incidence of early recurrence of atrial arrhythmias (ERAAs) compared with the patients who were not administered BTPs. The efficacy of BTPs at preventing ERAAs was confirmed not only immediately after ablation, but also during the short period after AF ablation.

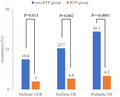

**Abbreviations**
AADs: antiarrhythmic drugs
AF: atrial fibrillation
AT: atrial tachycardia
BTP: bisoprolol transdermal patch
CB: cryoballoon
CIED: cardiac implantable electrophysiological devices
CTI: cavotricuspid isthmus
ECG: electrocardiogram
ERAA: early recurrences of atrial arrhythmias
LA: left atrium
MDCT: multidetector computed tomography
PV: pulmonary vein
PVI: pulmonary vein isolation
RF: radiofrequency
SVC: superior vena cava

## 1 INTRODUCTION

Atrial fibrillation (AF) ablation is an increasingly utilized and effective treatment option for patients with symptomatic AF. Early recurrences of atrial arrhythmias (ERAAs) often occur within the first 3 months of AF ablation, that is, during the “blanking period.” ERAAs are not associated with long‐term ablation failure,^1^ but they can be highly symptomatic and require therapeutic intervention. Although ERAAs may predict long‐term arrhythmia recurrences,^2^ the optimal medical therapy that prevents ERAAs during the blanking period requires clarification.

**FIGURE 1:**
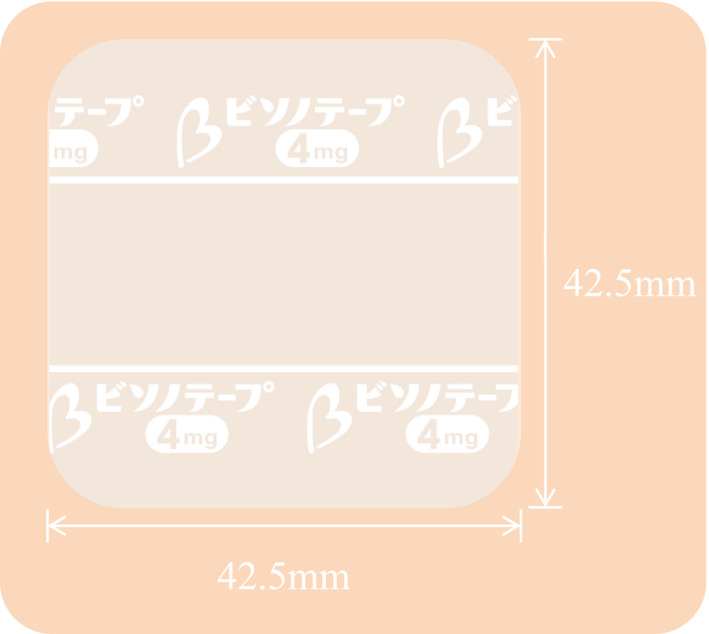
A bisoprolol transdermal patch, which is the world's first transdermal patch that contains the selective β1 blocker, bisoprolol

In January 2019, the world's first bisoprolol transdermal patch (BTP) (Bisono^®^ Tape; Toa Eiyo, Tokyo, Japan), which contains the selective β1 blocker, bisoprolol, became available for the treatment of tachycardia related to AF in Japan (Figure 1). As a transdermal drug delivery system, BTP is associated with several advantages compared with oral bisoprolol, including more stable concentrations of the drug in the plasma and reduced likelihoods of rapid changes in the blood pressure and heart rate.^3^, ^4^ Moreover, removal of BTPs is easy if hypotension or bradycardia occurs,^4^ and they can be administered immediately after AF ablation, even if a patient's arousal from anesthesia is delayed. The efficacy of BTPs at preventing ERAAs after ablation, however, has not been reported; therefore, we aimed to investigate whether BTPs prevent ERAAs in patients after ablation.

## 2 METHODS

### 2.1 Patients

**FIGURE 2:**
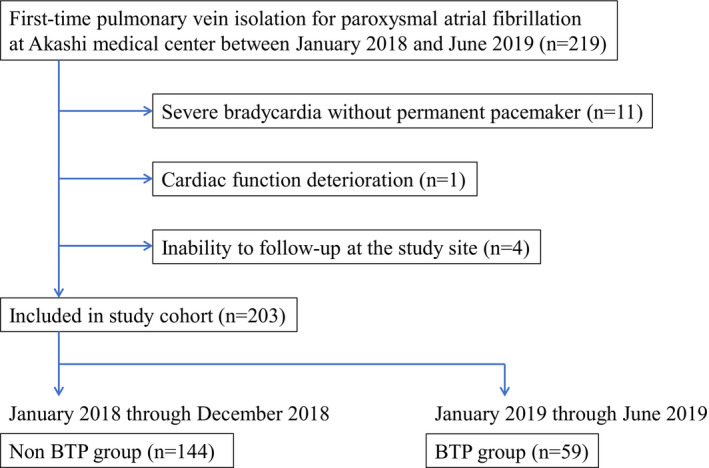
Flow diagram of the study's population. BTP, bisoprolol transdermal patch

This was a single‐center retrospective study of patients who underwent AF ablation at Akashi Medical Center. Between January 2018 and June 2019, 219 patients underwent their first ablation for paroxysmal AF. We excluded patients with poor cardiac function, which was defined as a left ventricular ejection fraction <35%, severe bradycardia without a permanent pacemaker, including atrioventricular block types two and three, sinoatrial blocks, and sick sinus syndrome, concomitant inflammatory conditions, including active infections, inflammatory arthritis, and connective tissue disease, malignancies, and those who were dependent on hemodialysis or unable to attend follow‐up assessments at the study site. After excluding 16 patients, the final analysis included 203 patients (Figure 2). From January 2019 when BTPs have become available for the treatment of AF, our institution routinely administered BTPs in the catheter laboratory immediately after ablation to prevent ERAAs unless there were contraindications. As a result, 59 patients were administered BTPs and they were enrolled into the BTP group. On the other hand, the patients who underwent ablation between January 2018 and December 2018 were assigned into the non‐BTP group.

This study was approved by the Akashi Medical Center's Ethics Committee, and it was performed in accordance with the tenets of the Declaration of Helsinki.

### 2.2 Consent

Informed consent was obtained in the form of opt‐out on the website.

### 2.3 Antiarrhythmic drug administration

Until December 2018, physicians could use antiarrhythmic drugs (AADs) after AF ablation to avoid the incidence of ERAAs during the blanking period. The type of AAD used, the dosage, and the timing of the administration were at the operating physician's discretion. BTPs have become available for the treatment of AF since January 2019, and our institution routinely administers BTPs in the catheter laboratory immediately after ablation to prevent ERAAs. The use of BTP was continued for 3 months after AF ablation, and, if possible, other AADs were discontinued before AF ablation. Although the BTP dosage was essentially 4 mg, which is equivalent to 2.5 mg of bisoprolol fumarate, it could be adjusted based on a patient's age, cardiac function, renal function, body weight, blood pressure, and heart rate.

### 2.4 Catheter ablation

All patients underwent contrast‐enhanced multidetector computed tomography (MDCT) to evaluate their left atria (LAs) and pulmonary veins (PVs). Three‐dimensional MDCT images of the LAs and PVs were reconstructed on a separate workstation and integrated using the NavX (St. Jude Medical, St. Paul, MN, USA), CARTO (Biosense Webster, Inc, Irvine, CA, USA), or Rhythmia (Boston Scientific Corporation, Cambridge, MA, USA) electro‐anatomical mapping systems.

PV isolation (PVI) was performed using 28‐mm second‐generation cryoballoon (CB) ablation, 28‐mm fourth‐generation CB ablation, or radiofrequency (RF) ablation. RF ablation was performed using an irrigated‐tip catheter with the maximum temperature set at 50˚C and a power output of 20‐35 W. The PVI endpoint was the creation of an entrance conduction block into the PV. After PVI, we performed the provocation of non‐PV triggers by the administration of isoproterenol and/or atrial burst pacing. When non‐PV triggers were not identified, additional ablation except for cavotricuspid isthmus (CTI) ablation was not performed. In contrast, when non‐PV triggers were identified with reproducibility, the initiation of AF or atrial tachycardia (AT) was routinely targeted on ablation. Then, a linear ablation, superior vena cava (SVC) isolation, and/or non‐PV trigger ablation were performed if additional ablation was needed. When triggers from the LA posterior wall were identified, posterior box was performed, creating a roof line and bottom line. If SVC triggers were identified, SVC isolation was performed. When non‐PV triggers form other sites were identified, focal ablation was applied around the earliest ectopic site. If macroreentrant AT was present, the mechanisms were determined by electro‐anatomical activation mapping or entrainment mapping, and linear ablation targeting the critical isthmus was performed. If the AF did not dissipate or was inducible after these procedures, sinus rhythm was restored by transthoracic cardioversion.

### 2.5 Follow‐up

After the procedures, the patients remained hospitalized under continuous rhythm monitoring (dynascope DS‐8900; Fukuda Denshi, Tokyo, Japan). After discharge, all patients attended monthly follow‐up assessments for 3 months. At each hospital visit, the patients underwent 12‐lead electrocardiography (ECG) examination and they were interviewed about any arrhythmia‐related symptoms, namely palpitations, chest discomfort, dizziness, and nausea. Holter ECG was performed at the physician's discretion between 1 month and 3 months after AF ablation. Moreover, for patients who had cardiac implantable electrophysiological devices (CIEDs), including pacemakers, implantable cardioverter‐defibrillators, cardiac resynchronization therapy devices, and loop recorders, we checked for AF recurrences using the CIEDs.

An early recurrence (ER) was defined as any episode of AF or AT that lasted ≥30 s and occurred within 90 days of AF ablation. We defined a very ER (VER) as a recurrence within 7 days of the AF ablation. VERs and ERs were classified as “definite” or “probable.” A definite VER or ER classification required electronic confirmation using an ECG monitor, 12‐lead ECG, Holter ECG, or a CIED. Probable VERs or ERs were defined as any arrhythmia‐related symptoms that were not confirmed electronically.

### 2.6 Statistical analyses

All data are presented as means and standard deviations (SDs) or proportions. The variables were compared using the Chi‐square or Fisher's exact tests, as appropriate. Kaplan‐Meier analysis was performed to assess recurrence‐free survival, and the log‐rank test was used to compare the groups. We performed a univariate analysis to identify potential factors associated with ERAAs, including clinical, medications, and ablation procedures. After a univariate analysis, all variables with *P*‐value <.1 were entered en bloc into the multivariate model along with age and sex as background variables. Thereafter, we used the stepwise multiple regression logistic analysis to explore the influence of different variables on ERAAs and to adjust for covariates. All of the analyses were performed using IBM^®^SPSS^®^ software, version 26 (IBM Corporation, Armonk, NY, USA), and a value of *P* < .05 was considered statistically significant.

## 3 RESULTS

### 3.1 Patients’ characteristics

The non‐BTP group comprised 144 consecutive patients with paroxysmal AF whose mean age was 66.7 ± 10.0 years, and it included 108 men (75%); this group of patients underwent their first AF ablation between January and December 2018. The BTP group comprised 59 consecutive patients with paroxysmal AF whose mean age was 66.9 ± 12.7 years, and it included 38 men (64.4%); this group of patients underwent their first AF ablation and received BTPs between January and June 2019. Table 1 presents the patients’ baseline characteristics. The groups did not differ regarding their epidemiological data, comorbidities, echocardiographic and computed tomography parameters, and laboratory data.

**TABLE 1:** Baseline characteristics

	Non‐BTP group (n = 144)	BTP group (n = 59)	*P* value
Epidemiological background			
Age, years	66.7 ± 10.0	66.9 ± 12.7	.91
Age ≥75 years (n, %)	36 (25)	14 (23.7)	.85
Male (n, %)	108 (75)	38 (64.4)	.13
Body mass index (kg/m^2^)	23.9 ± 3.6	23.5 ± 3.6	.45
Smoking (n, %)	73 (50.7)	28 (47.5)	.76
Duration of AF (months)	13.4 ± 21.2	10.9 ± 14.1	.41
Comorbidities (n, %)			
Hypertension	71 (49.3)	30 (50.8)	.842
Diabetes mellitus	22 (15.3)	9 (15.3)	.99
Hyperlipidemia	35 (24.3)	20 (33.9)	.16
Stroke	9 (6.3)	4 (6.8)	.55
Heart failure	4 (2.8)	2 (3.4)	.56
Coronary artery disease	8 (5.6)	6 (10.2)	.19
Valvular heart disease	11 (7.6)	5 (8.5)	.52
Dilated cardiomyopathy	1 (0.7)	0 (0)	.71
Hypertrophic cardiomyopathy	1 (0.7)	1 (1.7)	.50
Pacemaker	6 (4.2)	3 (5.1)	.51
ICD/CRT	1 (0.7)	0 (0)	.71
ILR	6 (4.2)	0 (0)	.12
Echocardiographic parameters			
LA‐diameter (mm)	35.6 ± 5.3	36.4 ± 4.6	.31
LV ejection fraction (%)	65.9 ± 6.9	66.0 ± 6.5	.90
Computed tomography			
LA volume (mL)	91.3 ± 2.1	91.0 ± 3.3	.94
LCPV (%)	18 (12.5)	7 (11.9)	.55
Laboratory data			
BUN (mg/dL)	16.0 ± 0.3	17.2 ± 0.6	.08
Creatinine (mg/dL)	0.88 ± 0.17	0.89 ± 0.31	.96
eGFR (ml/min/1.73 m^2^)	64.8 ± 1.2	63.7 ± 1.8	.59
Hemoglobin (mg/dL)	13.8 ± 0.11	13.8 ± 0.21	.20
BNP (pg/mL)	64.9 ± 8.1	99.2 ± 19.7	.11

Note

Values are expressed as mean ± SD.

Abbreviations: BTP, bisoprolol transdermal patch; AF, atrial fibrillation; ICD, implantable cardioverter‐defibrillator; CRT, cardiac resynchronization therapy; ILR, implanted loop recorders; LA, left atrium; LV, left ventricular; LCPV, left common pulmonary vein; BUN, blood urea nitrogen; eGFR, estimated glomerular filtration rate; BNP, brain natriuretic peptide.

### 3.2 Treatments and ablation procedures

Table 2 summarizes the patients' treatments and ablation procedures. The patients in the non‐BTP group received oral β‐blockers, including bisoprolol fumarate, carvedilol and atenolol (49.3%), flecainide (11.1%), bepridil (2.8%), and amiodarone (0.7%) during the study. All patients in the BTP group were administered BTPs immediately after AF ablation, of whom 58 (98.3%) received 4 mg bisoprolol fumarate as BTPs and one (1.7%) was administered 2 mg as a BTP. One in the BTP group was administered amiodarone.

**TABLE 2:** Medication during study and ablation procedure

	Non‐BTP group (n = 144)	BTP group (n = 59)	*P* value
Medication on discharge			
β‐blocker	71 (49.3)	59 (100)	<.0001
Oral bisoprolol fumarate	47 (32.6)	0 (0)	<.0001
Carvedilol	22 (15.3)	0 (0)	.001
Atenolol	2 (1.4)	0 (0)	.5
BTP	0 (0)	59 (100)	<.0001
Verapamil	2 (1.4)	0 (0)	.50
Bepridil	4 (2.8)	0 (0)	.25
Flecainide	16 (11.1)	0 (0)	.003
Amiodarone	1 (0.7)	1 (1.7)	.50
ACEi/ARB	48 (33.3)	20 (33.9)	.94
Statin	31 (21.5)	21 (35.6)	.037
Catheter ablation (n, %)			
CB	95 (66.0)	30 (50.8)	.044
The success of PVI	144 (100)	59 (100)	
CTI ablation	130 (90.3)	53 (89.8)	.92
Additional procedure^a^	25 (17.4)	11 (18.6)	.83
Posterior Box	17 (11.8)	6 (10.2)	.74
Roof line	5 (3.5)	3 (5.1)	.42
Bottom line	1 (0.7)	2 (3.4)	.20
Mitral isthmus line	1 (0.7)	1 (1.7)	.50
SVC isolation	2 (1.4)	0 (0)	.50
Non‐PV trigger ablation^b^	3 (2.1)	1 (1.7)	.67
Ablation procedure			
Total duration of procedure (min)	138.6 ± 39.3	147.3 ± 72.7	.17

Note

Values are expressed as mean ± SD.

Abbreviations: BTP, bisoprolol transdermal patch; ACEi, angiotensin‐converting enzyme inhibitor; ARB, angiotensin‐receptor blocker; RF, radiofrequency; CB, cryoballoon; PVI, pulmonary vein isolation; CTI, cavotricuspid isthmus; Posterior Box, roof line and additional posterior inferior line connecting the lower margins of right and left PV lines; SVC, superior vena cava.

^a^ Additional procedure is ablation procedures including posterior box, roof line, bottom line, mitral isthmus line, SCV isolation, and non‐PV trigger ablation except PVI and CTI ablation.

^b^ Non‐PV trigger ablation is elimination of non‐PV trigger by means of focal ablation at the site of origin except SVC isolation and posterior box.

Side effects presumed to be related to the BTPs led to the removal of patches in five patients (8.5%). The side effects comprised application‐site pruritus (n = 2), dizziness (n = 2), and leg edema (n = 1). The two patients with pruritus were switched to oral bisoprolol fumarate, and the remaining patients were administered other drugs. Serious adverse events, for example, clinically significant bradycardia, hypotension, and cardiac failure, were not observed. ERAAs were not observed during the study period among the patients who stopped using BTPs.

All patients' PVIs were successful, and entrance conduction blocks into PVs were created at all PV sites. CTI lines of the block with confirmations of bidirectional blocks were successfully created in 53 patients (89.8%) in the BTP group and 130 patients (90.3%) in the non‐BTP group. There were no differences between the groups regarding linear ablation, SVC ablation, or non‐PV trigger ablation, except that the use of CB ablation in the BTP group was less than that in the non‐BTP group (50.8% vs 66.0%). Internal cardioversion after AF ablation to restore sinus rhythm was performed on 27 patients (45.8%) in the BTP group and 63 patients (43.8%) in the non‐BTP group. The groups did not differ regarding the total procedure time (BTP and non‐BTP groups: 147.3 ± 72.7 min and 138.6 ± 39.3 min, respectively). Moreover, the groups did not differ regarding the total duration of hospital stay under continuous rhythm monitoring after AF ablation (BTP and non‐BTP groups: 3.51 ± 0.88 days and 3.71 ± 1.68 days, respectively).

### 3.3 Clinical outcomes

**FIGURE 3:**
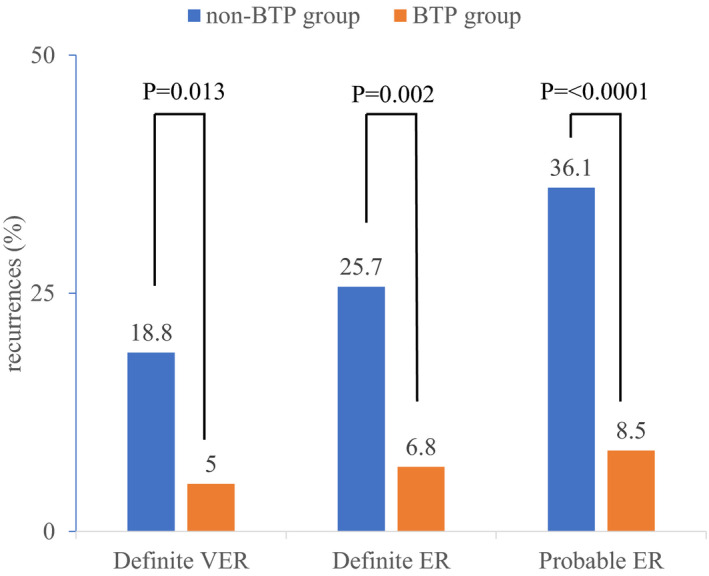
The incidence of early recurrences. BTP, bisoprolol transdermal patch; VERs, very early recurrences; ERs, early recurrences

**FIGURE 4:**
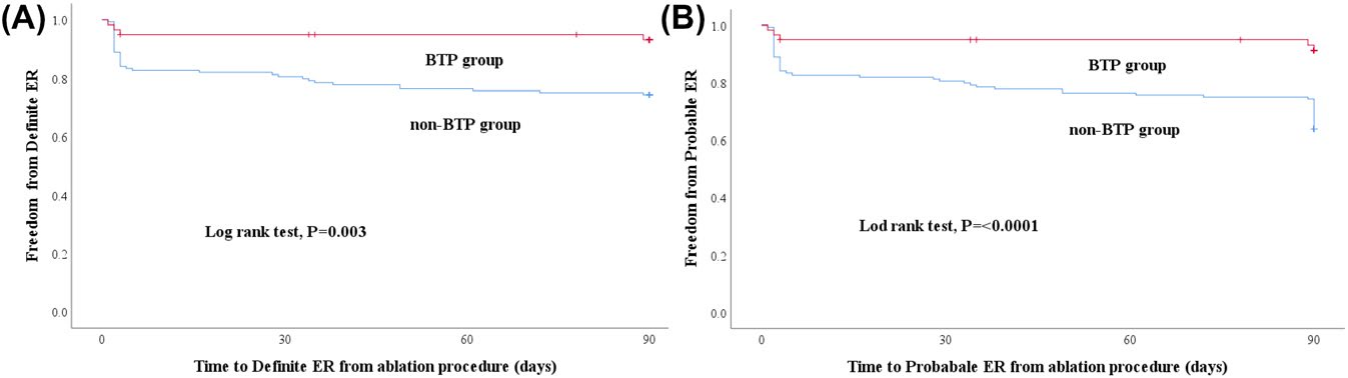
Kaplan‐Meier curves of the (A) freedom from definite early recurrences (ERs) and (B) freedom from probable ERs. Survival salvage analysis showed that the cumulative freedom from ERs was significantly lower in the bisoprolol transdermal patch (BTP) group than in the non‐BTP group

Figure 3 illustrates the incidence of ERAAs in the BTP and non‐BTP groups. During the 7‐day postablation period, VERs were observed significantly less frequently in the BTP group (n = 3; 5.0%) than in the non‐BTP group (n = 27; 18.8%) (*P* = .013). During the 3‐month postablation period, that is, the blanking period, fewer patients in the BTP group experienced definite ERs (n = 4; 6.8%) than those in the non‐BTP group (n = 37; 25.7%) (*P* = .002). Probable ERs were detected less frequently in the BTP group (n = 5; 8.5%) than in the non‐BTP group (n = 52; 36.1%) (*P* < .0001) during the blanking period. Kaplan‐Meier analysis showed a significant difference between the cumulative hazard curves in relation to definite ERs during the 3‐month postablation period (log‐rank test: *P* = .003) (Figure 4A). The mean recurrence‐free period was longer in the BTP group (85.5 days, [95% confidence interval (CI) 80.6‐90.4 days]) than that in the non‐BTP group (71.0 days, [95% CI 65.4‐76.6 days]). Kaplan‐Meier analysis showed a significant difference between the cumulative hazard curves for probable ERs during the 3‐month postablation period (log‐rank test: *P* < .0001) (Figure 4B). The mean recurrence‐free period was longer in the BTP group (85.5 days, [95% CI 80.0‐91.0 days]) compared with that in the non‐BTP group (71.0 days, [95% CI 65.3‐76.7 days]). All subjects without events were censored at 90 days.

In addition, we analyzed the comparison of baseline characteristics, medications, and ablation procedures between patients with or without definite ERs as Table 3. BTP was administered significantly less frequently in patients with definite ER (n = 4; 9.8%) than in those without definite ERs (n = 55; 34%) (*P* = .002). However, the administration of oral bisoprolol fumarate, carvedilol, or AADs was not significantly different between patients with and without definite ERs.

**TABLE 3:** Baseline characteristics and medication during study and ablation procedure between patients with or without definite ERs

	Definite ERs (‐) (n = 162)	Definite ERs (+) (n = 41)	*P* value
Epidemiological background			
Age, years	66.6 ± 10.0	67.4 ± 13.6	.68
Male (n, %)	116 (71.6)	30 (73.2)	.84
Body mass index (kg/m^2^)	24.0 ± 3.5	22.9 ± 3.1	.085
Duration of AF (months)	12.9 ± 19.6	12.0 ± 19.1	.78
Comorbidities (n, %)			
Hypertension	82 (50.6)	19 (46.3)	.63
Diabetes mellitus	26 (16)	5 (12.2)	.54
Hyperlipidemia	47 (29)	8 (19.5)	.22
Heart failure	5 (3.1)	1 (2.4)	.65
Pacemaker	6 (3.7)	3 (7.3)	.26
ICD/CRT	1 (0.6)	0 (0)	.80
ILR	3 (1.9)	3 (7.3)	.12
Echocardiographic parameters			
LA‐diameter (mm)	36.0 ± 5.2	35.2 ± 4.5	.38
LV ejection fraction (%)	65.9 ± 7.1	65.8 ± 5.4	.93
Laboratory data			
BNP (pg/mL)	69.2 ± 85.4	114.8 ± 83.7	.049
Medication on discharge			
Oral bisoprolol fumarate	37 (22.8)	10 (24.4)	.83
Carvedilol	18 (11.1)	4 (9.8)	.53
Atenolol	1 (0.6)	1 (2.4)	.37
BTP	55 (34)	4 (9.8)	.002
Bepridil	3 (1.9)	1 (2.4)	.6
Flecainide	11 (6.8)	5 (12.2)	.2
Amiodarone	2 (1.2)	0 (0)	.64
Catheter ablation (n, %)			
CB	102 (63)	23 (56.1)	.42
CTI ablation	150 (92.6)	33 (80.5)	.027
Additional procedure^a^	28 (17.3)	8 (19.5)	.74

Note

Values are expressed as mean ± SD.

Abbreviations: AF, atrial fibrillation; ICD, implantable cardioverter‐defibrillator; CRT, cardiac resynchronization therapy; ILR, implanted loop recorders; LA, left atrium; LV, left ventricular; BNP, brain natriuretic peptide; BTP, bisoprolol transdermal patch; CB, cryoballoon; CTI, cavotricuspid isthmus.

^a^ Additional procedure is ablation procedures including posterior box, roof line, bottom line, mitral isthmus line, SCV isolation, and non‐PV trigger ablation except PVI and CTI ablation.

Multivariate analysis was performed to identify independent risk and protective factors associated with ERAAs (Table 4). Following the univariate analysis that identified potential factors associated with ERAAs, including medications, comorbidities, and laboratory variables, the multivariate model was adjusted for age, sex, BTPs, the body mass index, statins, and CTI ablation. Administering BTPs was an independent factor that protected from ERAAs (odds ratio 0.181, [95% CI 0.059‐0.559], *P* = .003).

**TABLE 4:** Univariate and multivariate analyses of definite early recurrences after catheter ablation

	P value	OR	95% CI		P value	OR	95% CI
Univariate analysis				Multivariate analysis			
BTP	0.005	0.21	0.071‐0.620	BTP	0.003	0.181	0.059‐0.599
BMI	0.085	0.907	0.812‐1.014	BMI	0.033	0.886	0.793‐0.990
Statin	0.077	0.432	0.170‐1.097				
CTI ablation	0.025	0.33	0.125‐0.871	CTI ablation	0.010	0.250	0.087‐0.717

Abbreviations: OR, odds ratio; CI, confidence interval; BTP, bisoprolol transdermal patch; BMI, body mass index; CTI, cavotricuspid isthmus.

## 4 DISCUSSION

To the best of our knowledge, this is the first investigation into the effects of BTPs on the prevention of ERAAs. The study generated several novel findings that showed the incidence of ERAAs was significantly lower in the BTP group than that in the non‐BTP group, and that BTP might be a safe and effective means of preventing ERAAs during the 3‐month period immediately after ablation.

Although AF ablation primarily for PVI is a safe and effective therapeutic option for patients with paroxysmal AF, ERAAs are common problems during the early postablation period. A pooled analysis of several studies by Andrade et al^5^ showed that the incidence of ERAAs within the 3‐month period after RF catheter ablation across multiple studies ranged from 16% to 67%, with a mean pooled estimate of approximately 38%. In general, ERAAs during the blanking period were not considered to indicate ablation failure. However, some investigators have shown that ERAAs are highly indicative of late recurrences in studies that involved relatively large numbers of patients.^2^ Moreover, Xue et al^6^ showed that VERs were strongly associated with late recurrences. Hence, we considered that early treatment postablation was particularly important to prevent ERAAs.

To date, several strategies for preventing ERAAs have been described that include using AADs and anti‐inflammatory medications.^7^, ^8^, ^9^ Some investigators have suggested that using AADs during the blanking period might reduce the incidence of ERAAs and, thus, hospitalization and the need for cardioversion during the postablation period.^7^, ^8^, ^10^ Although administering AADs immediately after AF ablation reduced the incidence of ERAAs, it could not reduce the incidence of late recurrences from the antiarrhythmics after ablation of AF (5A) study^7^, ^8^. Sohns et al^11^ demonstrated that AADs were not superior to β‐blockers at preventing ERAAs, and that none of the AADs were superior to others in relation to maintaining sinus rhythm during the early postablation period. These investigators suggested that given their safety, β‐blockers might become an alternative to AADs for preventing ERAAs during the blanking period. Therefore, we aimed to investigate whether BTPs prevent ERAAs in patients after ablation. Transdermal drug delivery has several advantages; for example, hepatic first‐pass metabolism never occurs, the drug's action can be stopped quickly by removing the patch, if necessary, and the level of the drug in the blood can be maintained. During the perioperative period, BTPs are superior to oral bisoprolol for many reasons: compared with administering bisoprolol orally, the concentration of the drug in the plasma is more stable if it is administered transdermally, which may prevent rapid blood pressure and heart rate changes.^3^, ^4^ Moreover, if hypotension or bradycardia occurs, BTPs can be removed easily.^3^ After cardiac and/or thoracic aortic surgery, BTPs were more effective at reducing the incidence of postoperative AF than orally administered bisoprolol fumarate.^12^ Nonetheless, the efficacy of BTPs has not been described in the context of ablation. Immediately after surgery, patients are often unstable, and may become hypotensive or bradycardic. We selected BTPs as the pharmacological treatment for patients soon after AF ablation based on our reasoning that administering a drug with less potential to rapidly change the blood pressure and heart rate as soon as possible after ablation was desirable.

In the current study, the patients who were administered BTPs immediately after AF ablation showed significant reductions in the incidence of ERAAs compared with the patients who were not administered BTPs. The efficacy of BTPs at preventing ERAAs was confirmed not only immediately after ablation, but also during the short period after AF ablation without serious side effects. Hence, administering BTPs soon after AF ablation may be an effective and safe means to prevent ERAAs after AF ablation.

The mechanisms underlying ERAAs require full elucidation, but they could include inflammation during the acute phase after AF ablation, incomplete PVI, or the recovery of conduction in a previously isolated PV, non‐PV triggers of AF, and a transient imbalance of the autonomic nervous system.^1^, ^9^, ^13^ Many studies have shown that anti‐inflammatory medications, namely corticosteroids or colchicine, reduce ERAAs.^9^ These anti‐inflammatory medications had adverse effects, including hyperglycemia, infections, and gastrointestinal bleeding.^14^ Several investigators have reported that β‐blockers might have anti‐inflammatory effects and may attenuate the levels of inflammatory cytokines in heart failure, acute myocardial infarction, or dilated cardiomyopathy.^15^ Landiolol controls the production of cytokines and other inflammatory regulators that may also help to prevent AF and reduce postoperative complications^16^; therefore, the anti‐inflammatory effects of BTPs may contribute to the prevention of ERAAs.

Several reports described β‐blockers may have a relation to ectopic beats triggering AF and can suppress PV triggers.^17^ Furthermore, β‐blockers significantly suppress focal firing arising from the PV and non‐PV sites, even in combination with a sodium channel‐blocking agent.^18^ We consider that β‐blockers cannot directly prevent PV reconnection, but may reduce the incidences of ERAAs by suppressing PV triggering from the PV reconnection site and non‐PV triggers of AF.

β‐blockers may prevent excessive sympathetic nervous system activity. For example, the activation of the sympathetic nervous system plays an important role in the pathophysiology of AF postoperatively, and β‐blockers prevent postoperative AF by inhibiting sympathetic tone.^19^ The 2017 European guidelines recommend perioperative oral β‐blocker therapy as a class Ⅱa indication to prevent postoperative AF. Regarding ablation, O'Donnell et al and other investigators have suggested that increased levels of circulating catecholamines may cause ERAAs.^8^, ^20^ A strong association exists between sympathetic activation and AF recurrences.^21^ BTPs can provide a constant release of the drug for long periods of time. A previous study conducted to compare the BTP and oral bisoprolol fumarate using heart rate variability demonstrated that BTP had a more stable sympatholytic effect and more decreased autonomic fluctuation compared to oral bisoprolol fumarate^4^; therefore, we speculated that the administering BTP as soon as possible after ablation might reduce the incidences of ERAAs by preventing excessive sympathetic nervous system activity, followed by that the efficacy might be more lasting compared to an oral bisoprolol.

To summarize, the findings from the current study showed that by administrating BTPs immediately after AF ablation in the catheter laboratory, ERAAs might be suppressed as soon as possible compared with oral β‐blockers. ERAAs might be highly indicative of late recurrences; therefore, reducing ERAAs with BTPs could, theoretically, reduce late recurrences. Further follow‐up studies are needed to confirm this.

### 4.1 Limitations

The results of our study should be interpreted in the context of its limitations. First, this was a retrospective single‐center study that involved a small sample size, which might have led to the existence of statistical bias and confounding factors such as BMI and CTI ablation. Randomized controlled studies are required to confirm the effectiveness of BTPs at preventing ERAAs after ablation. Second, some patients may have had silent AF/AT recurrences and did not discuss these with a physician. Although we did not routinely perform Holter ECG during the blanking period, Holter ECG was performed in 91 patients (63.2%) in the non‐BTP group and 37 patients (62.7%) in the BTP group between 1 month and 3 months after AF ablation. However, even if patients received Holter ECG, we cannot deny the possibility of underdetection of ERAAs, because patients who had undergone the implantation of CIED were approximately 10% of all patients. The possibility of the underdetection of ERAAs was one of the limitations. Third, there was lack of uniform treatments and ablation procedure. AADs and/or β‐blockers were not administered to all patients in the non‐BTP group; however, BTPs were administered to all patients in the BTP group. Although oral bisoprolol fumarate, carvedilol, and AADs were not independent factors that protected from ERAAs by the univariate analysis, we cannot deny the possibility that lack of uniform treatments between the BTP and non‐BTP groups might affect the results. CB ablation was significantly more often performed in the non‐BTP group compared with BTP group. Although the multivariate analysis investigated that the CB was not an independent protective factor against the incidence of ERAAs, there was a possibility that the differences of ablation procedures had affected the incidences of ERAAs after ablation. In addition, we cannot deny the possibility of the influence of learning curve of ablation procedure. However, in almost cases of our study, well‐experienced physicians basically performed ablation. Even when inexperienced physicians performed ablation, the instructors were fixed during study period. Moreover, the instructors were not changed between the BTP and non‐BTP groups. As a result, there was no significant difference in the number of years of experience of the physicians between the BTP and non‐BTP groups. Fourth, although the blood levels of bisoprolol might be stable in the patient with BTP than those with oral bisoprolol, we did not measure the blood levels of bisoprolol for each patient. Fifth, we could not reveal the mechanism that BTP was superior to oral β‐blockers for the prevention of ERAAs. Finally, we could not determine the impact of BTPs on long‐term improvements in this study, therefore further studies are warranted to evaluate whether the long‐term use of BTPs reduces late clinical events.

## 5 CONCLUSION

The incidence of ERAAs after ablation in patients with paroxysmal AF was lower in the BTP group compared with that in the non‐BTP group. BTP might be useful for preventing ERAAs after ablation.

## CONFLICTS OF INTEREST

The authors declare no conflict of interest for this article.
